# A Bioluminescence Reporter Assay for Retinoic Acid Control of Translation of the GluR1 Subunit of the AMPA Glutamate Receptor

**DOI:** 10.1007/s12035-019-1571-9

**Published:** 2019-04-10

**Authors:** Thabat Khatib, Andrew Whiting, David R. Chisholm, Christopher Redfern, Berndt Müller, Peter McCaffery

**Affiliations:** 1 School of Medicine, Medical Sciences and Nutrition, Institute of Medical Sciences, University of Aberdeen, Foresterhill, Aberdeen, Scotland AB25 2ZD UK; 2 Department of Chemistry, Durham University, South Road, Durham, DH1 3LE UK; 3 Northern Institute for Cancer Research, Medical School, Newcastle University, Newcastle upon Tyne, NE2 4HH UK

**Keywords:** RAR, Retinoic acid, GluR1, Non-genomic

## Abstract

Retinoic acid (RA) regulates numerous aspects of central nervous system function through modulation of gene transcription via retinoic acid receptors (RARs). However, RA has important roles independent of gene transcription (non-genomic actions) and in the brain a crucial regulator of homeostatic plasticity is RAR control of glutamate receptor subunit 1 (GluR1) translation. An assay to quantify RAR regulation of GluR1 translation would be beneficial both to study the molecular components regulating this system and screen drugs that influence this critical mechanism for learning and memory in the brain. A bioluminescence reporter assay was developed that expresses firefly luciferase under the control of the GluR1 5′ untranslated region bound by RAR. This assay was introduced into SH-SY5Y cells and used to demonstrate the role of RARα in RA regulation of GluR1 translation. A screen of synthetic RAR and RXR ligands indicated that only a subset of these ligands activated GluR1 translation. The results demonstrate the practicality of this assay to explore the contribution of RARα to this pathway and that the capacity of RAR ligands to activate translation is a quality restricted to a limited number of compounds, with implications for their RAR selectivity and potentially their specificity in drug use.

## Introduction

Nuclear receptors are involved in a major set of signalling pathways in the brain, and crucial among these is the retinoic acid receptor (RAR) family [1]. They have a well described mechanism of action to regulate gene expression [2]. In addition, “non-genomic” roles have been described for these receptors and a vital action for retinoic acid receptor alpha (RARα) in the brain is regulation of mRNA translation during homeostatic synaptic plasticity [3].

Synaptic connections in the brain are highly plastic. The number and strength of synapses in neural pathways can be modified in response to different factors such as experience and this is an important element in the formation of memory. With changes in synaptic strength, however, it is important to maintain the stability of neural networks in order to prevent the neural circuits from becoming hyper- or hypo-active [4, 5]. The ability of neurons to adjust their activity levels and maintain a balance between the relative strength of individual synapses is called homeostatic synaptic plasticity [6, 7]. Homeostatic synaptic plasticity adjusts total synaptic strength through different mechanisms such as regulating the release and/or reuptake of presynaptic transmitters and changing the number and/or sensitivity of postsynaptic receptors in order to maintain a balance [8, 9]. For example, pharmacological manipulation studies have shown that an increase in synaptic strength is induced when neural activity is inhibited by tetrodotoxin (TTX; which blocks sodium-gated voltage channels) and an *N*-methyl-D-aspartate receptor (NMDA) receptor antagonist [10, 11]. This synaptic increase in neural activity is mediated by an increase in local translation and insertion of the glutamate receptor 1 (GluR1, also known as GRIA1 and GluA1) subunits of the α-amino-3-hydroxy-5-methyl-4-isoxazolepropionic acid (AMPA) receptor [12–14]. AMPA receptors are glutamate excitatory transmembrane receptors that mediate most of the neuroexcitatory synaptic transmission in the central nervous system. AMPA receptors are composed of four subunits (GluR1-GluR4), and they mediate neuroplasticity and play an important role in cognition, memory and learning [15].

Retinoic acid (RA) is synthesised in neurons in response to a decrease in neural activity. Aoto and colleagues demonstrated that the application of RA to hippocampal cultures increased the amplitude of spontaneous excitatory postsynaptic currents and that activity blockade induced RA synthesis in the neurons [16–18]. The synthesised RA mediated a type of homeostatic plasticity by regulating the translation of the GluR1 subunit of the AMPA receptor in postsynaptic membranes [3]. It does this through a cytoplasmically localised population of the nuclear receptor RARα [16]. In the absence of RA, the F-domain of RARα binds directly to consensus sequences in the 5′ untranslated region (5′ UTR) of GluR1 mRNA; this is thought to inhibit the scanning mechanism, whereby the 43S pre-initiation complex searches for the initiation codon to start translation. During synaptic scaling, blockade of synaptic activity triggers RA synthesis and the RA binds to RARα. The ligand binding domain of RARα undergoes a conformational change shifting position of helix 12, and it is proposed also the adjacent F-domain [3], resulting in the weakening of the affinity for mRNA; and so the GluR1 mRNA is released allowing it to be translated. This causes an increase in the postsynaptic AMPA receptor levels [3, 16].

In the current study, we wanted to design a simple quantitative system that detects the effects of RA analogues on post-transcriptional gene regulation in neurons during synaptic scaling, in order to study factors that can promote or inhibit this regulatory system. A bioluminescence reporter plasmid that expresses firefly luciferase under the control of the GluR1 5′ UTR region was produced and introduced into SH-SY5Y neuroblastoma cells, a cell line commonly used as a model of neuronal differentiation and function [19]. RARα was either overexpressed or knocked down in these cells to demonstrate its involvement in RA control of GluR1 translation. Synthetic retinoids were then tested in this assay for their ability to regulate AMPA receptor translation. We demonstrate the utility of this system for screening RAR ligands for their therapeutic capacity to promote neuroplasticity and for elucidating the molecular components that regulate this form of translational regulation.

## Materials and Methods

### Retinoids

**Fig. 1:**
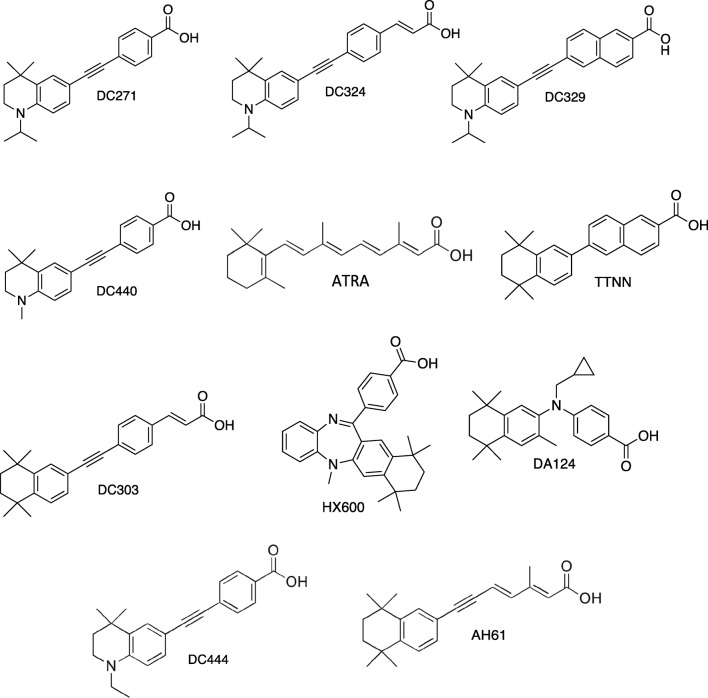
Synthetic retinoids used for screening

All-*trans* RA (ATRA) was purchased from Sigma-Aldrich. HX600 and DA124 were a gift from Dr. Kagechika (Tokyo). Synthetic retinoids and some non-retinoid homologues were designed and prepared by the Whiting group (Durham University) as described previously [20–24]. The molecular structure of the RAR and RXR ligands used is shown in Fig. 1.

### Construction of Plasmids for GluR1 Assay

Firefly luciferase GluR1 reporter plasmid pTK1 was built from a pGL3 (R2.1) vector (Promega) derivative that expresses luciferase destabilised by addition of a PEST motif under the control of a simian virus 40 (SV40) early promoter and is referred to here as pGL3 (R2.1) promoter.

To generate pTK1, a 309-bp 5′ UTR fragment of the rat GluR1 gene was generated by PCR from genomic DNA prepared from rat HCN-A94 neural stem cells, using the Expand™ High Fidelity PCR System kit (Sigma-Aldrich) and primers AGGAGAGCAGAGGGAGAGG and CAAAGATGTACGGCATATTCCTT. Fifteen base-pair extensions homologous to the vector ends were added by PCR using nested primers CTTTTGCAAAAAGCTTGCTCGGCTCCCCTTCC and TTGGCGTCTTCCATGGAGATTTGGTCTTCCCTCCCC. The resulting PCR products were then inserted into HindIII/NcoI cut pGL3 (R2.1) promoter vector using the In-Fusion® HD Cloning Kit (Clontech) to generate GluR1 recombinant plasmid pTK1 containing the rat GluR1 5′ UTR. The sequence was confirmed by Sanger sequencing performed at the Dundee University DNA sequencing service.

### CRISPR/CAS9 Plasmid Construction for Knockout of RARα

The Gibson Designer tool at the Wellcome Trust Sanger Institute Genome Editing database (WGE) [25] was used to identify exon 7 as a target for disruption of the human RARα open reading frame (ORF). Exon 7 was chosen because it is present in all transcripts, and removing it causes frameshift mutations leading to premature termination codons that make the RNA subject to nonsense-mediated decay. Guide sequences in RARα exon 7 were identified using the CRISPR Finder tool in WGE. Oligonucleotides CACGCGGTACACGCCCGAGC and GCTCGGGCGTGTACCGCGTG were annealed in CutSmart® Buffer (NEB) and cloned into the BbsI cut pX330-U6-Chimeric_BB-CBh-hSpCas9 plasmid (Addgene 42,230) using Instant Sticky-end Ligase Master Mix (NEB) to produce pTK3.

A homologous direct repair (HDR) donor plasmid was prepared in order to introduce a puromycin cassette to repair the DNA double-strand break generated by Cas9 nuclease in RARα exon 7. The plasmid contains a puromycin selection marker flanked by 1253 and 1313 base pairs of genomic DNA from upstream and downstream of RARα exon 7. These fragments were amplified from genomic SH-SY5Y DNA using primers CATGTGAGGCAAGAGATAAGTCAAC and CTGAACCCGAACCCACTCTGAG, and TCTGTTAGGTATCTCTAGAGGGCAG and GCATCTTTCTTGGGATTCAGTTCTT, respectively, using Phusion® High-Fidelity DNA Polymerase (NEB). Further PCR reactions were then carried out to add extension to the 5′ and 3′ flanks of the PCR products that were homologous to the vector ends using primers AAACGACGGCCAGTGAATTCCATGTGAGGCAAGAGATAAGTCAAC and GCCGTTTGGTTCGAAGTTCCCTGAACCCGAACCCACTCTGAG, and TATCATGTCTGGATCCGGGGTCTGTTAGGTATCTCTAGAGGGCAG and CCATGATTACGCCAAGCTTGATGCATCTTTCTTGGGATTCAGTTCTT, respectively. The puromycin cassette was amplified from SERP_100_puc19_GIBSON_EF1a_PURO plasmid (a gift from Dr. Bill Skarnes, Wellcome Trust Sanger Institute) using primers GGAACTTCGAACCAAACGGC and CCCCGGATCCAGACATGATA. The plasmid backbone was obtained by digesting SERP_100_puc19_GIBSON_EF1a_ PURO plasmid with EcoRI/ HindIII enzymes. The four overlapping DNA fragments were joined together to produce pTK4 using the Gibson Assembly® Cloning Kit (NEB) according to the manufacturer’s recommendations.

### Cell Lines and Cell Culture Conditions

SH-SY5Y cells [26] were grown in T-75 culture flasks in DMEM (Thermo Fisher Scientific) containing 10% FCS (Thermo Fisher Scientific) and at 5% CO_2_/37 °C. SH-SY5Y cells (SH-SY5Y^RARα^) in which human RARα cDNA was conditionally over-expressed in response to tetracycline were supplied by Dr. Danielle Lindley [27]. These cells had been prepared by stable transfection of human RARα2 cDNA (Mammalian Gene Collection) into SH-SY5Y^tet12^ cells (parental SH-SY5Y cells transfected with the pcDNA6/TR tet repressor plasmid [28]) and clonally-selected using methods described by Goranov et al. [29]. SH-SY5Y^RARα^ cells were cultured in T-75 culture flasks in DMEM containing 10% FCS, 5 μg/ml blasticidin (Invitrogen) and 250 μg/ml zeocin (Invitrogen) antibiotics to maintain selection of the tet repressor and RARα plasmid constructs respectively. For all cell cultures, the medium was changed three times a week, and the cells were passaged at about 70% confluence using 0.05% trypsin-EDTA solution. The passage number used for each experiment was no higher than 30.

### Preparation of RARα^±^ Hemizygous Knockout SH-SY5Y Line

SH-SY5Y cells were plated in 6-well plates at a density of 1.2 × 10^6^ cells per well in 1-ml media and left to attach overnight in DMEM medium containing 10% FCS. Cells were then transfected with 1.5 μg of pTK3 and pTK4 plasmids each using jetPRIME® transfection reagent (Polyplus-transfection SA) according to the manufacturer’s recommendation. Cells were then incubated for 4 h in a 5% CO_2_/37 °C incubator before exchanging with normal DMEM containing 10% FCS without puromycin antibiotic. The next day, the medium was replaced with DMEM with 10% FCS and containing 2.5 μg/ml puromycin for selection. The medium was changed every 3 days for a 10-day period.

Single SH-SY5Y colonies were picked and grown to confluency in wells of a 24-well plate containing 500 μl of DMEM with 10% FCS and 2.5 μg/ml puromycin. The cells in each well were then split and some of them were used for DNA analysis while the rest were grown for cryopreservation. DNA was extracted from SH-SY5Y cells using an ISOLATE genomic DNA mini Kit (Bioline) according to the manufacturer’s instructions. PCR reactions were carried out to confirm RARα gene disruption using a GoTaq G2 polymerase (Promega) to analyse the RARα gene using five different pairs of primers.

**Fig. 2:**
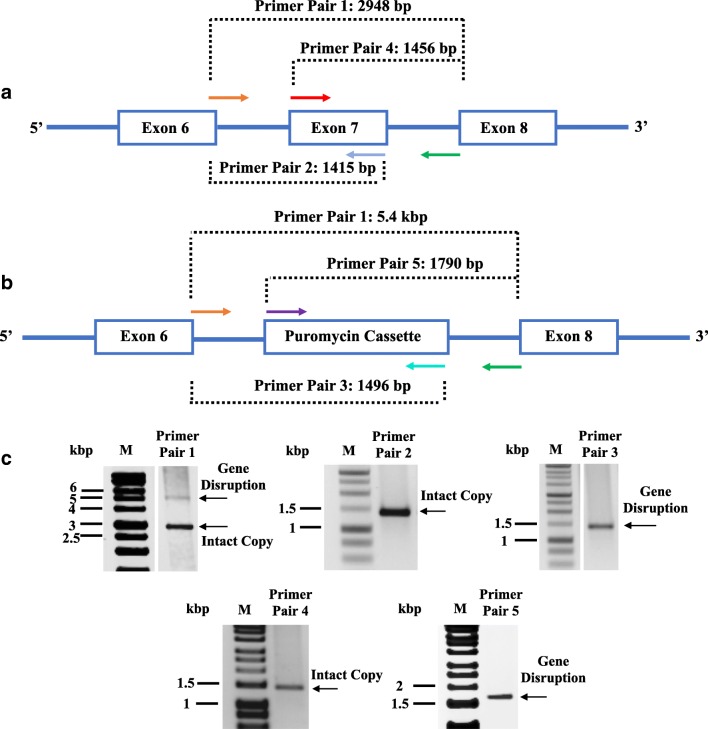
Analysis of RARα gene disruption. **a** Schematic drawing of the RARα wild type gene structure showing the region between exon 6 and exon 8. **b** RARα gene structure after CISPR-Cas9-mediated replacement of exon 7 by the puromycin expression cassette. The diagnostic PCR amplicons produced using different pairs of primers are shown. Primer pairs 2 and 4 are specific for the wild type RARα gene while primer pairs 3 and 5 detect the insertion of the puromycin expression cassette. Primer pair 1 spans the region containing exon 7 and produces a longer product when the puromycin expression cassette has been inserted. **c** Characterisation of a hemizygous RARα^±^ cell line. Genomic DNA isolated from a clonal SH-SY5Y cell line was analysed using the primer combinations described above, and amplicons were analysed by 1% agarose gel electrophoresis. Both puromycin cassette and exon 7 were detected in the clonal SH-SY5Y cells which indicate that only one copy of RARα was knocked out. M is a DNA size standard

The location of primer pairs used to characterise the knockout cell lines is shown in Fig. 2. Primer pair 1 (TGCACCACCATCCCCTCTCT and CGAGGTGGGAAGATCAATACTGCT) binds upstream and downstream of the region used for gene disruption (exon 7 deletion) and normally produces a 2.9-kbp product. Insertion of the puromycin cassette increases the length of this amplicon to 5.4 kbp. Primer pairs 2 (TGCACCACCATCCCCTCTCT and CCGAAGCCAGCGTTGTG) and 4 (TCAGCGCCATCTGCCTCAT and CGAGGTGGGAAGATCAATACTGCT) both detect the presence of exon 7, and both produce amplicons of 1.4 kbp. Insertion of the puromycin cassette at this locus is detected by primer pairs 3 (TGCACCACCATCCCCTCTCT and GCATGCTCTTCTCCACCTCAGT) and 5 (CTTCACCGTCACCGCCG and CGAGGTGGGAAGATCAATACTGCT) and they produce amplicons of 1.5 kbp and 1.8 kbp, respectively.

### GluR1 Translation Assay

SH-SY5Y cells were seeded in 24-well plates at a density of 20,000 cells per well in 500 μl DMEM containing 10% FCS without antibiotics. SH-SY5Y cells were then transfected with one of the recombinant reporter plasmids pTK1 using jetPRIME® transfection reagent. In parallel, cells were transfected with two control plasmids pGL3 (R2.1) promoter and the pGL3-Basic plasmid (Promega, GenBank U47295) without promoter. In addition, each recombinant/control plasmid was co-transfected with a plasmid expressing *Renilla* luciferase (pRL-TK; Promega; GenBank AF025846.2) used as an internal transfection control. The transfection mixtures were prepared using 600 ng of recombinant/control firefly plasmid and 150 ng of *Renilla* luciferase plasmid pRL-TK and added to cells according to the manufacturer’s recommendation,

Twenty-four hour after transfection, the medium was replaced with normal culture medium containing the test retinoid or DMSO alone as a standard. Retinoid dilutions were prepared in DMEM with 10% FCS from stock solutions in DMSO. Each retinoid was tested in triplicate at 10 μM concentration with a final DMSO concentration of 0.1%. In the case of SH-SY5Y^RARα^ cells, the cells were also treated with doxycycline (Melford) at a concentration of 1 μg/ml along with the retinoids to drive RARα overexpression.

### Dual Luciferase Assay

Firefly and *Renilla* luciferases were measured sequentially using the Dual-Luciferase reporter assay system (Promega) according to the manufacturer’s protocol. After 24 h of retinoid treatment, the medium was removed and the cells were washed with 250 μl of PBS; 100 μl of passive lysis buffer was then added to each well, and the plates incubated on a plate shaker at room temperature for 15 min. Cell lysates (10 μl) were transferred into wells of a 96-well white luminometer plate (Greiner Bio-One), and the luciferase assays were performed using a GloMax® 96 Microplate Luminometer (Promega) according to the manufacturer’s protocol.

### Protein Analysis and Western Blotting

Cells were washed with cold PBS and lysed with 150 mM NaCl, 1% Triton, 0.1% SDS, 50 mM HEPES containing protease inhibitor cocktail (Sigma) in double-distilled water. Protein concentrations were measured using a BCA assay kit (Thermo Fisher Scientific). Fifty micrograms of protein was loaded and separated by electrophoresis through 12% SDS-polyacrylamide gels and transferred to nitrocellulose membranes. Rabbit anti-RARα primary antibody (Santa cruz, sc-551) along with horseradish peroxidase-conjugated secondary anti rabbit IgG antibody (Jackson Immunoresearch) and anti-β-actin peroxidase mouse primary antibody (Sigma, A3854) were used in the study. Western blots were developed using enhanced chemiluminescence (Millipore), and the protein bands were detected and scanned using a myECL Imager (ThermoScientific).

### Statistical Analysis

All data are presented as mean ± SEM of three independent experiments. Statistical analyses were performed in Microsoft Office Excel 2017, GraphPad Prism 7.0c version (Prism, GraphPad Software, San Diego, CA) or R version 3.3.1 (R Core Team, Vienna, Austria: The R Foundation for Statistical Computing). The data were analysed by Student’s *t* test, one-way ANOVA with Newman-Keuls multiple comparison test, or linear models with RARα expression level as an ordered factor, as appropriate; a *p* value < 0.05 was considered statistically significant. *p ≤ 0.05, **p ≤ 0.01, ***p ≤ 0.001, ****p ≤ 0.0001.

## Results

### A Dual Luciferase Assay for Detecting RARα-Ligand Regulation of GluR1 Translation

**Fig. 3:**
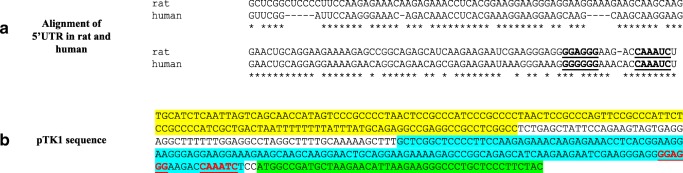
Retinoic acid control of GluR1 translation. **a** Alignment of 5′ UTR sequences of human and GluR1 mRNA. The RARα consensus binding sites identified previously by Poon and Chen using SELEX are underlined and in bold. **b** Partial sequence of the pTK1 construct showing the SV40 promotor shaded in yellow, the GluR1 5′ UTR shaded in blue, and the beginning of the firefly luciferase open reading frame in green. The RARα consensus binding sites are in red and underlined, while vector sequence is unshaded

To quantify RARα ligand regulation of GluR1 translation, a highly sensitive bioluminescence reporter composed of firefly luciferase under the control of the GluR1 5′ UTR was employed. The rat GluR1 5′ UTR sequence was chosen based on previous work by Poon and Chen who identified consensus RNA binding motifs in the 5′ UTR regions of rat GluR1 mRNA that preferentially bind RARα for translational control [3]. This region including the RARα binding motifs is conserved in the human GluR1 5′ UTR (Fig. 3a). Based on that, a firefly luciferase GluR1 reporter plasmid, designated pTK1, was designed by cloning a fragment of the 5′ UTR region (that contains consensus motifs) from rat GluR1 into a pGL3 (R2.1) under control of the SV40 promoter. Figure 3 b shows the sequence of the relevant regions of pTK1 construct.

**Fig. 4:**
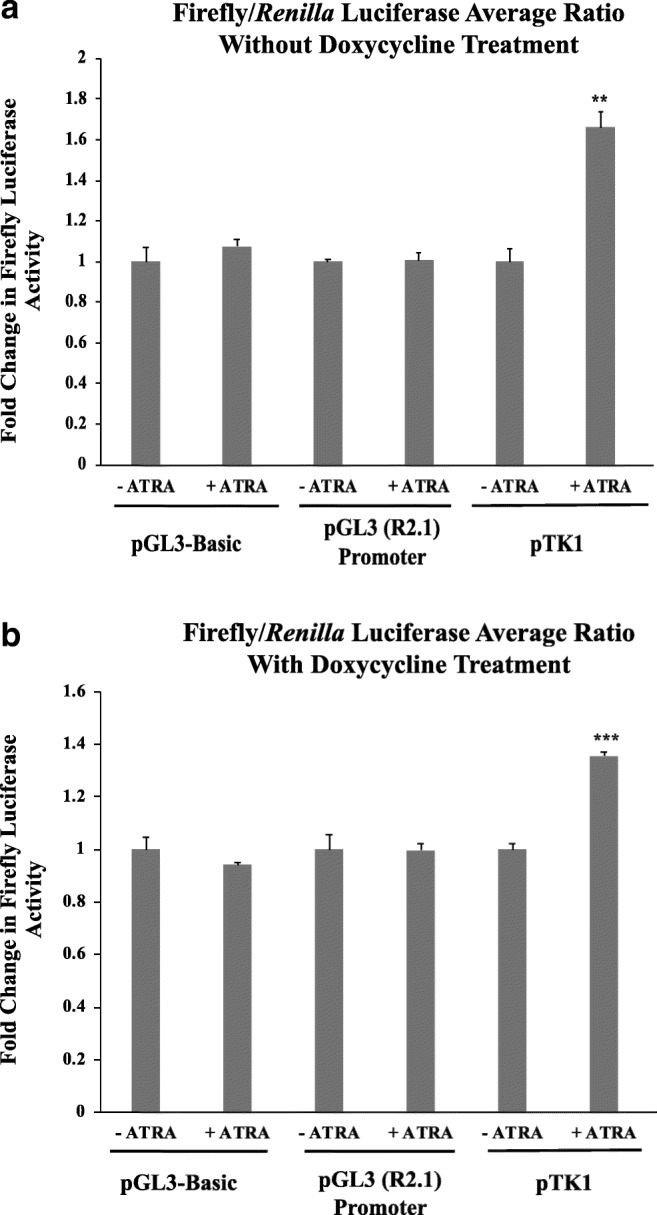
pTK1 containing the rat GluR1 5′ UTR region is sensitive to RA-treatment of SH-SY5Y cells. SH-SY5Y^RARα^ cells were transfected with the indicated plasmids and with pRL-TK expressing *Renilla* luciferase as a reference. After transfection, cells were treated with 10-μM ATRA for 24 h before luciferase activities were measured. **a** The graph shows standardised firefly luciferase activity in SH-SY5Y ^RARα^ cells grown in the absence of doxycycline. Firefly luciferase activity was normalised with respect to *Renilla* luciferase activity and then standardised with respect to normalised luciferase activity in cells not treated with ATRA (−ATRA), which was set at 1. **b**) Luciferase activity in SH-SY5Y ^RARα^ cells treated with doxycycline. Shown are normalised, standardised luciferase activities determined as described for **a**. Shown are mean values of three biological replicates. Error bars indicate standard error of the mean (SEM) (**p ≤ 0.01, ***p ≤ 0.001, Student’s *t* test)

The construct was transfected into SH-SY5Y cells, a frequently used model of neuronal cells, employing a SH-SY5Y^RARα^ variant in which high levels of RARα expression can be induced with tetracycline or the more stable derivative doxycycline [30]. SH-SY5Y^RARα^ cells were transfected with the rat GluR1 reporter pTK1, or a pGL3 (R2.1) promoter parental plasmid lacking the insert to demonstrate that the response is due to the sequence inserted. In addition, the pGL3-Basic plasmid, lacking a promoter but containing a normal firefly luciferase gene without the destabilising PEST sequence present in the pGL3 (R2.1) derivatives, was used as a sensitive control for promoter and GluR1 5′ UTR-independent effects of RARα on luciferase expression. For all assays, cells were co-transfected with pRL-TK expressing *Renilla* luciferase as reference and independent measure of transfection efficiency. Transfected cells were treated with 10-μM ATRA for 24 h, and then luciferase activities were measured (Fig. 4). Ten μM ATRA was used because in many studies SH-SY5Y cells are treated with this concentration of ATRA in order to study its action [31–34].

In a first experiment, the effect of ATRA on reporter gene expression was analysed in SH-SY5Y^RARα^ cells grown in the absence of doxycycline, and so without RARα overexpression (Fig. 4a). Firefly and *Renilla* luciferase activities were measured separately, and for comparison, firefly luciferase activity was normalised with respect to *Renilla* luciferase activity and standardised with respect to activity in cells not treated with ATRA. In the case of the two controls, pGL3-Basic and pGL2 (R2.1) promoter, luciferase activity did not significantly differ between cells treated with/without ATRA. On the other hand, cells transfected with pTK1 containing the rat GluR1 5′ UTR showed an approximately 1.6-fold increase in luciferase expression when treated with ATRA. These results indicate that the reporter assay was, as expected, responsive to RA.

The effect of RARα overexpression was tested by inducing RARα expression with doxycycline. Overexpression of RARα had no significant effect on the response to ATRA of the different plasmids used as negative controls: only luciferase under the control of the rat GluR1 5′ UTR region was increased by treatment with ATRA (Fig. 4b). However, given that the response to ATRA was significantly less following doxycycline-induced RARα over-expression (*p* = 0.0231), routine assays were done without doxycycline-treatment of SH-SY5Y^RARα^ cells.

### The Luciferase Assay Is Sensitive to RARα Levels

**Fig. 5:**
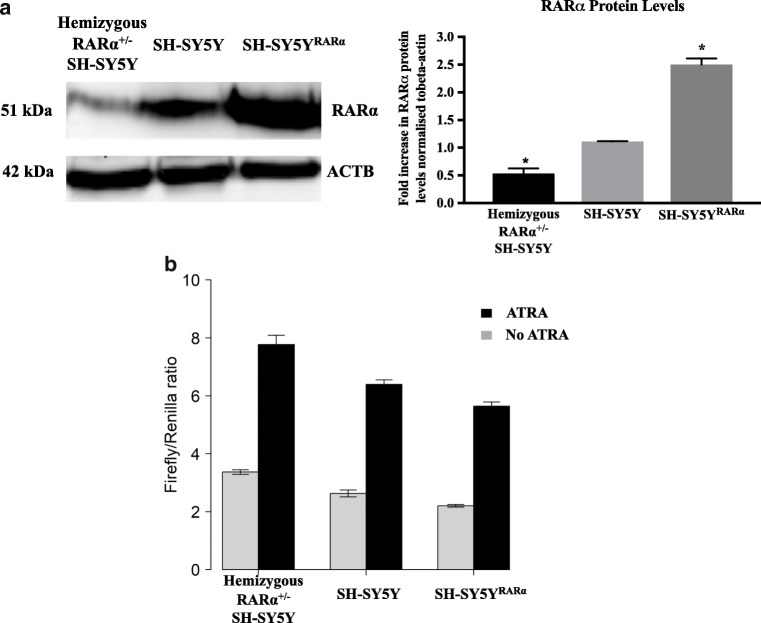
RARα levels influence the pattern of inhibition of translation. **a** A Western blot showing RARα levels in SH-SY5Y^RARα^ cells that overexpress the receptor after 1-μg doxycycline treatment for 24 h, SH-SY5Y cells and hemizygous RARα^±^ SH-SY5Y cells. The graph shows the ratio of RARα levels normalised to β-actin (ACTB) for all three cell types. Shown are mean values of three biological replicates. Error bars indicate standard error of the mean (SEM) (*p ≤ 0.05, one-way ANOVA with Newman-Keuls multiple comparison test). **b** The three different SH-SY5Y cell lines were transfected with pTK1 containing the rat GluR1 5′ UTR region and pRL-TK, and treated with/without 10-μM ATRA. After 24 h, cells were lysed and luciferase activity was measured. The firefly luciferase activity was normalised with respect to *Renilla* luciferase activity. Shown are the average of normalised luciferase levels (firefly/*Renilla*) of three biological replicates. Error bars indicate SEM. The data were analysed using a linear model with ATRA treatment and RARα expression (ordered factor) as explanatory variables (see text)

Our assay models the effects of RARα in controlling translation of GluR1 mRNA. To test whether the response was sensitive to RARα levels, we directly compared the response to ATRA in SH-SY5Y hemizygous RARα^±^ knockout cells to parental SH-SY5Y cells and SH-SY5Y^RARα^ cells overexpressing RARα after 1 μg doxycycline treatment. Analysis by Western blotting demonstrated clear differences in RARα protein levels in these three cell types (Fig. 5a), with a 2.5-fold higher RARα level in the doxycycline-treated SH-SY5Y^RARα^ cell line and a 2.1-fold lower RARα level in the hemizygous RARα^±^ SH-SY5Y cell line compared to the parental SH-SY5Y cells.

The three different cell types were then transfected with pTK1 and pRL-TK and treated with/without ATRA to determine the effects of RARα levels on the reporter assay (Fig. 5b). There was a clear reduction in reporter activity (effect coefficient − 0.8) as RARα expression levels increased (Linear model with RARα expression as an ordered factor: *p* < 0.001; Fig. 5b), and ATRA treatment had a clear effect in inducing a 2.3- to 2.6-fold increase in reporter activity (Linear model, effect of RA, *p* < 0.001). In addition, ATRA treatment had a significant effect (Linear model, interaction term, *p* = 0.04) in increasing the negative relationship between RARα expression levels and reporter activity. These results show that the effect of RARα is dose-dependent, with the lowest reporter activity in cells with the highest RARα expression (Fig. 5b).

**Fig. 6:**
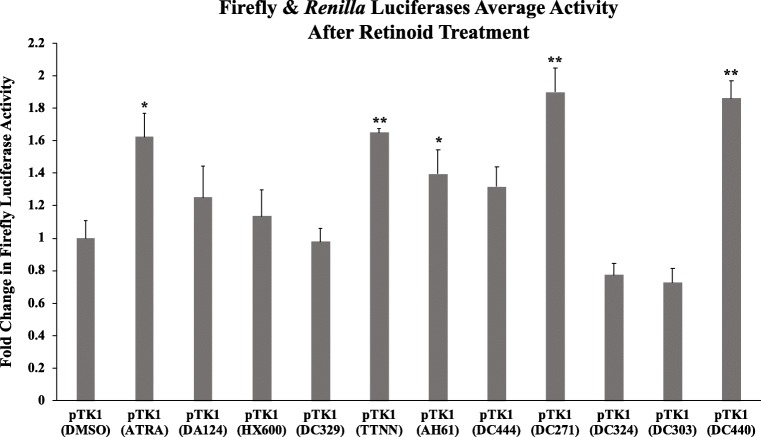
Identification of synthetic retinoids that act on the GluR1 5′ UTR region. SH-SY5Y^RARα^ were transfected with pTK1 containing the rat GluR1 5′ UTR region and with pRL-TK. After 24 h, they were treated with 10 μM of retinoids. After a further 24 h, cells were lysed and luciferase activities were measured as described. The graph shows firefly luciferase activity normalised with respect to *Renilla* luciferase activity, with activity in cells treated with DMSO only set at 1. Shown are mean values of three biological replicates. Error bars indicate SEM (*p ≤ 0.05; **p ≤ 0.01, one-way ANOVA with Newman-Keuls multiple comparison test)

Having established that pTK1 containing the rat GluR1 5′ UTR region was, as expected, responsive to RA, we exploited this to screen novel synthetic RAR and two commercially available RXR ligands with differing capacity to induce ligand-activated transcription (unpublished data), for their ability to regulate GluR1 translation compared to ATRA. TTNN, AH61, DC271, DC440 and DC444 are analogues of ATRA that exhibit strong binding affinity for the RARs, while DC303, DC324 and DC329 are non-active retinoid analogues that were designed specifically to be longer structures than RA and would therefore be unable to bind into the RAR ligand-binding pocket [21, 35, 36]. HX600 [37] and DA124 [38] are known to be RXR agonists that do not exhibit significant binding affinity for the RARs. SH-SY5Y^RARα^ cells cotransfected with pTK1 containing the rat GluR1 5′ UTR region, and pRL-TK expressing *Renilla* luciferase were treated for 24 h with 10 μM retinoids, and for comparison with 10 μM ATRA or with the solvent DMSO (Fig. 6). In addition to ATRA, treatment with only four out of the tested RAR or RXR ligands, namely TTNN, AH61, DC271 and DC440 caused a statistically significant increase in luciferase activity. In addition, treatment with DC444 also increased luciferase activity, but not significantly. DA124 and HX600 did not have any effect on luciferase activity as expected because they are RXR, and not RAR, ligands.

## Discussion

A sensitive bioluminescence-based dual luciferase assay was developed to enable the study of the translational control of GluR1 mRNA by RARα. For this assay, the 5′ UTR region of rat GluR1 gene was inserted upstream of the initiation codon of a luciferase gene. Using this construct, together with pRL-TK expressing *Renilla* luciferase as reference and in combination with SH-SY5Y cells with normal, elevated or reduced RARα levels, the effect of RARα levels, and of synthetic RAR/RXR ligands, could be quantified for their capacity to regulate GluR1 translation by measuring changes in luciferase activity.

Control of translation by RA via RARα is a non-genomic activity, breaking the dogmatic view of the RARs as purely transcriptional regulators restricted to the nucleus [3, 16, 39]. RARα is actively exported from the nucleus by a nuclear export signal (NES) present in domain E (the ligand binding domain) of the receptor [3, 40]. In the cytoplasm, RARα acts as an RNA binding protein, binding directly to different mRNAs such as dendritically localised GluR1 mRNA. The C-terminal F-domain of RARα binds to specific sequences in the 5′ UTR region of the rat GluR1 mRNA and represses the translation of GluR1 [3]. This mechanism of inhibition may be similar to the translational control of ferritin mRNA by iron regulatory protein (IRP), which binds to the ferritin mRNA and prevents the binding of the 43S pre-initiation complex to it [41]. RA binding to RARα causes conformational changes in the receptor which relieves the association between RARα and GluR1 mRNA, resulting in GluR1 translation [3]. Schwertz and colleagues also showed that RARα can regulate mRNA translation via interaction with the 3′ UTR of genes, as well as their 5′ UTR region [42]. They reported in human platelets the presence of the RARα consensus binding sites identified previously by Poon and Chen [3] in the 3′ UTR region of microtubule associated protein 1 light chain 3 beta 2 (MAP1LC3B2) transcripts, the 3′ UTR and 5′ UTR regions of SLAIN motif containing protein 2 (SLAIN2) and the 5′ UTR region of angiopoietin-1 (ANGPT1) [42]. Thus RA can regulate translation of several proteins [42] but GluR1 is the only regulated subunit presently described to be part of an ionotropic receptor.

Notably, this study demonstrated that RARα is involved in GluR1 translation as reported previously [3, 16, 39] by demonstrating a negative dose-dependent effect of RARα on the GluR1 translation assay (Fig. 5). However, we could not confirm whether RARα is the only receptor regulating GluR1 in response to RA, as we were unable to test cells lacking all RARα genes.

An ATRA concentration of 10 μM was used in this assay. Almost all studies treating SH-SY5Y cells with ATRA use a high, 10 μM concentration, for example [31–34]. The most effective concentration for inducing differentiation of SH-SY5Y cells using ATRA is 10 μM concentration [43, 44], which may be because ATRA rapidly induces strong expression of the catabolic enzymes CYP26A1 and CYP26B1 [45], which counteract the effects of RA, thus necessitating high concentrations.

A range of RAR ligands together with two known RXR ligands were tested for their capacity to regulate GluR1 translation (Fig. 6). Of those tested, only four increased GluR1 translation significantly, i.e. DC271, DC440, TTNN and AH61 with DC271 and DC440 also being stronger than ATRA, although not significantly. As expected, neither of the RXR ligands, DA124 nor HX600 increased GluR1 translation. Thus, it is the synthetic retinoids that activate RARs, and not those that only activate RXRs, which induce GluR1 translation. The use of the dual luciferase GluR1 translation assay to screen for bioactive compounds that promote GluR1 translation will identify routes by which homeostatic plasticity in the brain may be promoted. This may be of application to disorders such as fragile X syndrome in which homeostatic plasticity is impaired which may contribute to the neural dysfunction in this disease [46].

In summary, a reporter gene system has been developed to study the translational control of GluR1 by RA. The assay was used to investigate the influence of levels of RARα expression on GluR1 translation. This approach can be used to investigate any protein that influences this pathway as proposed for fragile X mental retardation protein (FMRP) [46]. Exploration of the system may show a level of control parallel to the enormous complexity of RAR regulated transcription and the dozens of coregulators that contribute to this. This assay can also be used to identify RARα ligands for their capacity to increase GluR1 expression which will then increase AMPA receptor levels and which may provide therapeutics that regulate homeostatic plasticity in the brain. The insights demonstrated here could potentially be used to design RAR ligands of greater specificity for RAR triggered pathways, allowing the development of RAR-based therapeutics with fewer side effects for disorders impacted on by neuroplasticity, such as Alzheimer’s disease [47].

## References

[1] Ana Maria A, Oscar Andréas M-R, Haider NB (2016). Role of nuclear receptors in central nervous system development and associated diseases. J Exp Neurosci.

[2] Aranda A, Pascual A (2001). Nuclear hormone receptors and gene expression. Physiol Rev.

[3] Poon MM, Chen L (2008). Retinoic acid-gated sequence-specific translational control by RAR. Proc Natl Acad Sci.

[4] Turrigiano GG, Nelson SB (2004). Homeostatic plasticity in the developing nervous system. Nat Rev Neurosci.

[5] Abbott LF, Nelson SB (2000). Synaptic plasticity: taming the beast. Nat Neurosci.

[6] Turrigiano GG, Leslie KR, Desai NS, Rutherford LC, Nelson SB (1998). Activity-dependent scaling of quantal amplitude in neocortical neurons. Nature.

[7] Burrone J, Murthy VN (2003). Synaptic gain control and homeostasis. Curr Opin Neurobiol.

[8] Davis GW (2006). Homeostatic control of neural activity: from phenomenology to molecular design. Annu Rev Neurosci.

[9] Turrigiano G (2012). Homeostatic synaptic plasticity: local and global mechanisms for stabilizing neuronal function. Cold Spring Harb Perspect Biol.

[10] Lissin DV, Gomperts SN, Carroll RC, Christine CW, Kalman D, Kitamura M, Hardy S, Nicoll RA, Malenka RC, von Zastrow M (1998). Activity differentially regulates the surface expression of synaptic AMPA and NMDA glutamate receptors. Proc Natl Acad Sci U S A.

[11] Watt AJ, Van Rossum MCW, MacLeod KM (2000). Activity coregulates quantal AMPA and NMDA currents at neocortical synapses. Neuron.

[12] Ju W, Morishita W, Tsui J, Gaietta G, Deerinck TJ, Adams SR, Garner CC, Tsien RY, Ellisman MH, Malenka RC (2004). Activity-dependent regulation of dendritic synthesis and trafficking of AMPA receptors. Nat Neurosci.

[13] Sutton MA, Ito HT, Cressy P, Kempf C, Woo JC, Schuman EM (2006). Miniature neurotransmission stabilizes synaptic function via tonic suppression of local dendritic protein synthesis. Cell.

[14] Sutton MA, Wall NR, Aakalu GN, Schuman EM (2004). Regulation of dendritic protein synthesis by miniature synaptic events. Science.

[15] Gouaux E (2004). Structure and function of AMPA receptors. J Physiol.

[16] Aoto J, Nam CI, Poon MM, Ting P, Chen L (2008). Synaptic signaling by all-trans retinoic acid in homeostatic synaptic plasticity. Neuron.

[17] Chen N, Onisko B, Napoli JL (2008). The nuclear transcription factor RARα associates with neuronal RNA granules and suppresses translation. J Biol Chem.

[18] Chen N, Napoli JL (2008). All- trans -retinoic acid stimulates translation and induces spine formation in hippocampal neurons through a membrane-associated RARα. FASEB J.

[19] Nicolini G, Miloso M, Zoia C, di Silvestro A, Cavaletti G, Tredici G (1998). Retinoic acid differentiated SH-SY5Y human neuroblastoma cells: an in vitro model to assess drug neurotoxicity. Anticancer Res.

[20] Haffez H, Chisholm DR, Valentine R, Pohl E, Redfern C, Whiting A (2017). The molecular basis of the interactions between synthetic retinoic acid analogues and the retinoic acid receptors. Med Chem Comm.

[21] Clemens G, Flower KR, Gardner P, Henderson AP, Knowles JP, Marder TB, Whiting A, Przyborski S (2013). Design and biological evaluation of synthetic retinoids: probing length vs. stability vs. activity. Mol BioSyst.

[22] Christie VB, Barnard JH, Batsanov AS, Bridgens CE, Cartmell EB, Collings JC, Maltman DJ, Redfern CPF, Marder TB, Przyborski S, Whiting A (2008). Synthesis and evaluation of synthetic retinoid derivatives as inducers of stem cell differentiation. Org Biomol Chem.

[23] Zhou G-L, Tams DM, Marder TB, Valentine R, Whiting A, Przyborski SA (2013). Synthesis and applications of 2,4-disubstituted thiazole derivatives as small molecule modulators of cellular development. Org Biomol Chem.

[24] Haffez H, Chisholm DR, Tatum NJ, Valentine R, Redfern C, Pohl E, Whiting A, Przyborski S (2018). Probing biological activity through structural modelling of ligand-receptor interactions of 2,4-disubstituted thiazole retinoids. Bioorg Med Chem.

[25] Hodgkins A, Farne A, Perera S, Grego T, Parry-Smith DJ, Skarnes WC, Iyer V (2015). WGE: a CRISPR database for genome engineering. Bioinformatics.

[26] Biedler JL, Helson L, Spengler BA (1973). Morphology and growth, tumorigenicity, and cytogenetics of human neuroblastoma cells in continuous culture. Cancer Res.

[27] Lindley D (2010) Retinoic acid receptor expression and activation and cellular responses to retinoic acid in neuroblastoma. Unpublished PhD thesis, Newcastle University.

[28] Lovat PE, Oliverio S, Ranalli M, Corazzari M, Rodolfo C, Bernassola F, Aughton K, Maccarrone M, Hewson QD, Pearson AD, Melino G, Piacentini M, Redfern CP (2002). GADD153 and 12-lipoxygenase mediate fenretinide-induced apoptosis of neuroblastoma. Cancer Res.

[29] Goranov BB, Campbell Hewson QD, Pearson ADJ, Redfern CPF (2006). Overexpression of RARγ increases death of SH-SY5Y neuroblastoma cells in response to retinoic acid but not fenretinide. Cell Death Differ.

[30] Gossen M, Freundlieb S, Bender G, Muller G, Hillen W, Bujard H (1995). Transcriptional activation by tetracyclines in mammalian cells. Science (80- ).

[31] Forster JI, Köglsberger S, Trefois C, Boyd O, Baumuratov AS, Buck L, Balling R, Antony PMA (2016). Characterization of differentiated SH-SY5Y as neuronal screening model reveals increased oxidative vulnerability. J Biomol Screen.

[32] Dwane S, Durack E, Kiely PA (2013). Optimising parameters for the differentiation of SH-SY5Y cells to study cell adhesion and cell migration. BMC Res Notes.

[33] Xicoy H, Wieringa B, Martens GJM (2017). The SH-SY5Y cell line in Parkinson’s disease research: a systematic review. Mol Neurodegener.

[34] Cheung Y-T, Lau WK-W, Yu M-S, Lai CSW, Yeung SC, So KF, Chang RCC (2009). Effects of all-trans-retinoic acid on human SH-SY5Y neuroblastoma as in vitro model in neurotoxicity research. Neurotoxicology.

[35] Gluyas JBG, Burschka C, Dörrich S, Vallet J, Gronemeyer H, Tacke R (2012). Disila-analogues of the synthetic retinoids EC23 and TTNN: synthesis, structure and biological evaluation. Org Biomol Chem.

[36] Whiting A, Marder T (2014) Fluorescent synthetic retinoids. UK patent application no. GB1417957.6, 2014.

[37] Morita K, Kawana K, Sodeyama M, Shimomura I, Kagechika H, Makishima M (2005). Selective allosteric ligand activation of the retinoid X receptor heterodimers of NGFI-B and Nurr1. Biochem Pharmacol.

[38] Ohta K, Tsuji M, Kawachi E (1998). Potent retinoid synergists with a diphenylamine skeleton. Biol Pharm Bull.

[39] Maghsoodi B, Poon MM, Nam CI, Aoto J, Ting P, Chen L (2008). Retinoic acid regulates RARalpha-mediated control of translation in dendritic RNA granules during homeostatic synaptic plasticity. Proc Natl Acad Sci U S A.

[40] Huang H, Wei H, Zhang X, Chen K, Li Y, Qu P, Zhang X, Chen J, Liu Y, Yang L, Li T (2008). Changes in the expression and subcellular localization of RARα in the rat hippocampus during postnatal development. Brain Res.

[41] Gray NK, Hentze MW (1994). Iron regulatory protein prevents binding of the 43S translation pre-initiation complex to ferritin and eALAS mRNAs. EMBO J.

[42] Schwertz H, Rowley JW, Zimmerman GA, Weyrich AS, Rondina MT (2017). Retinoic acid receptor-α regulates synthetic events in human platelets. J Thromb Haemost.

[43] Shipley MM, Mangold CA, Szpara ML (2016) Differentiation of the SH-SY5Y human neuroblastoma cell line. J Vis Exp:1–11. 10.3791/53193 10.3791/53193 PMC4828168 26967710

[44] Encinas M, Iglesias M, Liu Y, Wang H, Muhaisen A, Ceña V, Gallego C, Comella JX (2002). Sequential treatment of SH-SY5Y cells with retinoic acid and brain-derived neurotrophic factor gives rise to fully differentiated, neurotrophic factor-dependent, human neuron-like cells. J Neurochem.

[45] Stoney PN, Fragoso YD, Saeed RB, Ashton A, Goodman T, Simons C, Gomaa MS, Sementilli A, Sementilli L, Ross AW, Morgan PJ, McCaffery PJ (2016). Expression of the retinoic acid catabolic enzyme CYP26B1 in the human brain to maintain signaling homeostasis. Brain Struct Funct.

[46] Zhang Z, Marro SG, Zhang Y, Arendt KL, Patzke C, Zhou B, Fair T, Yang N, Südhof TC, Wernig M, Chen L (2018). The fragile X mutation impairs homeostatic plasticity in human neurons by blocking synaptic retinoic acid signaling. Sci Transl Med.

[47] Chang PKY, Verbich D, Mckinney RA (2012). AMPA receptors as drug targets in neurological disease - advantages, caveats, and future outlook. Eur J Neurosci.

